# Biochemical study of the effect of mesenchymal stem cells-derived exosome versus l-Dopa in experimentally induced Parkinson’s disease in rats

**DOI:** 10.1007/s11010-023-04700-8

**Published:** 2023-03-26

**Authors:** Asmaa S. Mohamed, Dina S. Abdel-Fattah, Ghada A. Abdel-Aleem, Thanaa F. El-Sheikh, Manal M. Elbatch

**Affiliations:** 1 Present Address: Medical Biochemistry Department, Faculty of Medicine, Tanta University, El-Geish Street, Tanta, El Gharbia Egypt; 2 Medical Biochemistry Department, Faculty of Medicine, Cairo University, Giza, Egypt

**Keywords:** Parkinson’ disease (PD), Exosome, DJ-1, PARKIN, circRNA.2837, microRNA-34b

## Abstract

Parkinson’s disease (PD) is a chronic and ongoing neurological condition. Unfortunately, as the dopaminergic terminals continue to deteriorate, the effectiveness of anti-Parkinson therapy decreases. This study aimed to examine the effects of BM-MSCs-derived exosomes in rats induced with Parkinson’s disease. The goal was to determine their potential for neurogenic repair and functional restoration. Forty male albino rats were divided into four groups: control (group I), PD (group II), PD-l-Dopa (group III), and PD-exosome (group IV). Motor tests, histopathological examinations, and immunohistochemistry for tyrosine hydroxylase were performed on brain tissue. The levels of α-synuclein, DJ-1, PARKIN, circRNA.2837, and microRNA-34b were measured in brain homogenates. Rotenone induced motor deficits and neuronal alterations. Groups (III) and (IV) showed improvement in motor function, histopathology, α-synuclein, PARKIN, and DJ-1 compared to group (II). Group (IV) showed improvement in microRNA-34b and circRNA.2837 compared to groups (III) and (II). MSC-derived exosomes showed a greater suppression of neurodegenerative disease (ND) compared to l-Dopa in Parkinson’s patients.

**Graphical abstract:**

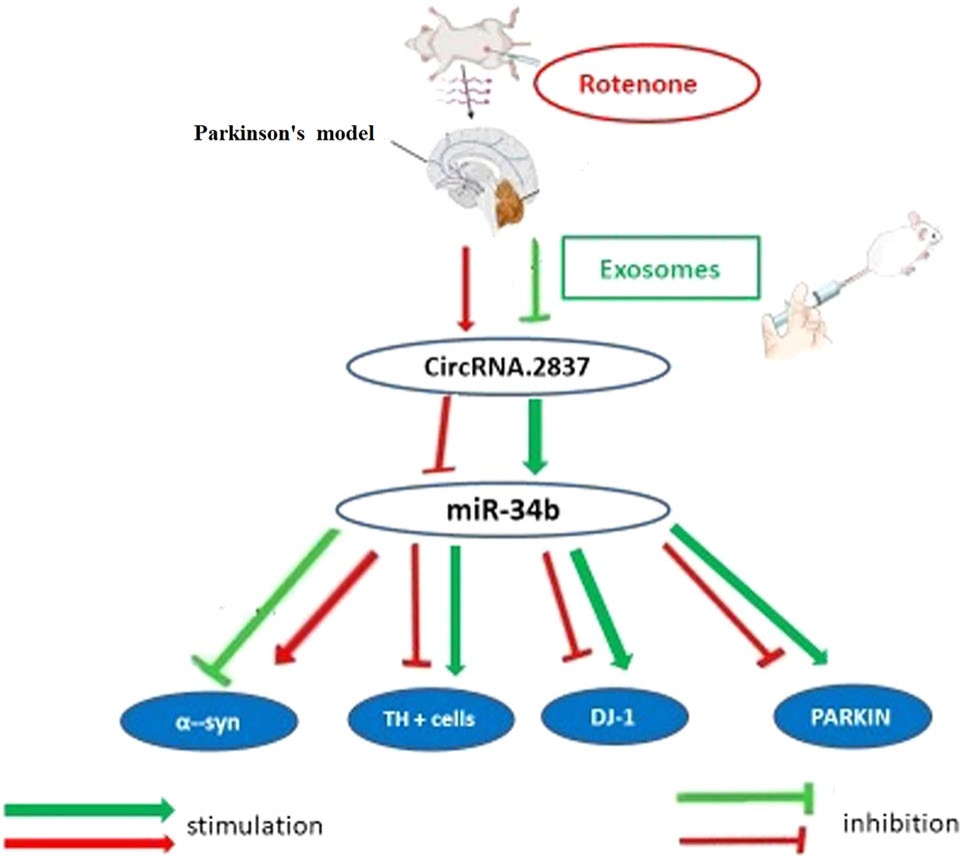

**Supplementary Information:**

The online version contains supplementary material available at 10.1007/s11010-023-04700-8.

## Introduction

Parkinson’s disease (PD) is a severe neurological disorder that impacts a large number of people all over the world. Despite advances in research, the exact cellular and molecular mechanisms involved in PD are still not understood. The current treatment options improve the quality of life for those with PD, but they are unable to stop its progression or increase the survival or differentiation of dopaminergic neurons [1]. The potential of MSC-derived secretome to enhance dopamine neuron longevity, induce neurogenesis, and reduce neuroinflammation has led to the exploration of MSC-derived secretome and its exosome derivatives as a new therapy for ND diseases, including PD [2].

The therapeutic results seen after MSC implantation were comparable to those produced by exosomes generated from MSCs. Exosomes produced from mesenchymal stem cells (MSCs) can prevent the unintended change in the MSCs and reduce the risk of an immune response from a different individual, which is often a concern with MSC-based treatments [3]. Furthermore, exosomes possess the capability to penetrate the blood–brain barrier, making them valuable as indicators of neurological disorders.

Exosomes can be retrieved from any type of bodily fluid and hold a diverse range of contents, including microRNAs, ribosomal RNAs, non-coding RNAs, lipids, protein molecules, and DNA. The components of these contents are determined by the tissue source of the exosomes. The proteinaceous molecules known as “exosome markers” participate in the role of exosomes and also include membrane molecules that can assist in identifying the biological origin of the exosomes, thereby improving their sensitivity as markers. In neurodegenerative diseases, such as Parkinson’s disease, exosomes contain misfolded molecules, such as alpha-synuclein. Of the various exosome-related biomolecules, microRNAs have received the most attention as markers [4].

MicroRNAs are a type of non-coding RNA that regulate multiple genes by impacting mRNA. Several microRNAs have been identified as modulators of α-synuclein. For example, increased levels of microRNA-16-1 decrease the translation of the HSP70 mRNA, which plays a role in inhibiting α-synuclein. Additionally, preventing microRNA-34b from binding to α-synuclein increases its levels. In Parkinson’s disease (PD), a correlation was found between the decrease of microRNA-34b and the subsequent decline of PARKIN and DJ-1 in various brain regions [5].

CircRNAs are types of ncRNAs. They play a crucial role in controlling gene expression through both transcription and post-transcriptional processes. These RNAs have been implicated in various human diseases. Researches have revealed that circRNAs are abundant in mammalian neural tissues and are strongly associated with the development and progression of neurodegenerative diseases. One such circRNA is circRNA.2837, which is commonly increased in sciatic nerve injury cases and acts as a microRNA sponge for the microRNA-34 family, thus inhibiting their activities [6].

## Materials and methods

### Materials

#### Chemicals

Rotenone was obtained from the Cornell Lab-Chemistry Company in Cairo, Egypt. Sinemet® tablets were purchased from Merck & Sharp & Dohme (MSD)—(Italia) S.P.A/Italy and imported by RAMCO. Each tablet contains 250 mg of l-Dopa and 25 mg of carbidopa.

#### Rats

This research was conducted on 40 male albino rats which were equally distributed into four groups (*n* = 10/group), weighing between 200 and 250 g. The rats were housed in clean, well-ventilated cages in the animal house. The environment was controlled, with a natural light/dark cycle, room temperature, and standard laboratory feeding and watering. The rats were given one week to acclimate to their new surroundings before the trial began. The study protocol was approved by the ethics committee at Tanta University in Egypt (Approval code 34014/8/20).

### Methods

#### Study design

**Fig. 1:**
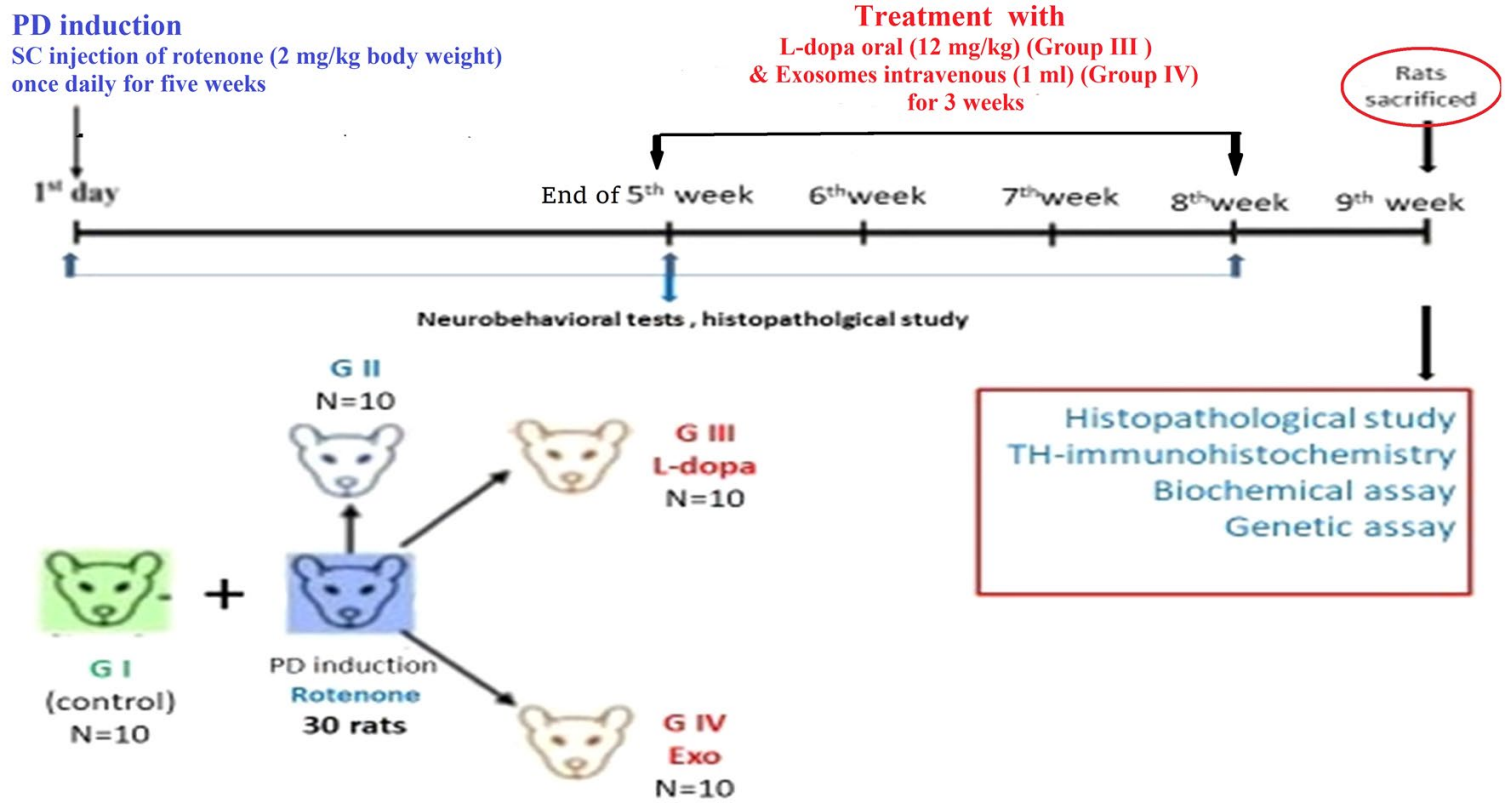
Flowchart of experimental design

This research was conducted in the Medical Biochemistry Department of Cairo University’s Faculty of Medicine, located in Egypt. Four groups of rats were established, each group included 10 rats, as shown in Fig. 1:

1. *Group I* (healthy controls = 10 rats) included rats that received a regular diet and were in good health.
2. *Group II* (pathological controls = 10 rats) consisted of rats with Parkinson’s disease (PD) induced through subcutaneous injection of rotenone (2 mg/kg body weight) dissolved in sunflower oil, administered once daily for 5 weeks (35 days) [7].
3. *Group III* (PD-l-Dopa = 10 rats) received the same dose of rotenone as those in group II. After PD induction (after 35 days of daily rotenone injection), these rats were given Sinemet® pills orally. The pills were dissolved in water and given orally twice daily for three consecutive weeks at a dosage of 12 mg/kg [8].
4. *Group IV* (PD-BM-MSC-derived exosome = 10 rats) received an equal amount of rotenone as those in group II. After PD induction, each rat received an intravenous dosage of 1 ml of prepared exosomes in the rat’s tail once weekly for three consecutive weeks [9, 10].

At the end of the experiment (1 week after the last dose of exosomes and l-Dopa), all animals’ groups were killed under anesthesia and brain tissue samples were collected and examined. Finally, killed rats were safely collected in a special package according to safety precautions and infection control measures and sent with the hospital biohazards.

#### Behavioral examinations

Throughout the duration of the experiment, motor and cognitive tests were conducted at three different stages to assess the progression of the disease: initially, following rotenone injections, and after treatment.

- Vertical Pole test
  As stated by Chompoopong et al., the pole test was utilized to examine the bradykinesia and agility of rats. The rats were placed on top of a vertical wooden pole with their heads facing upward. The time taken for orienting downward (turn time) and descending the wooden pole (total time) were assessed twice. Before receiving rotenone, the rats performed the pole test for 2 days [11].
- Morris Water Maze (MWM) test
  As stated by Vorhees and Williams, the MWM test was used to assess memory and cognitive ability. Briefly, the rats were placed in a pool of water colored with milk powder, where they swam to a concealed escape platform which remained there during training. The amount of time taken by the rats to climb the platform was recorded as the escape latency time. Twenty-four hours later, the platform was removed and the retention time was assessed, which is the time spent at the location where the platform previously existed. Before receiving rotenone injections, the rats were trained in the MWM for 2 days [12].

#### Isolation of bone marrow mesenchymal stem cells (BM-MSCs)

Male albino rats (7 weeks old and 150–200 g) were euthanized and slaughtered by cervical displacement. MSCs were isolated from the tibia and femur of the rats using phosphate-buffered saline (PBS). The procedure involved stacking 15 ml of flushed BM cells over 15 ml of Ficoll–Hypaque, followed by centrifugation at 400×*g* rpm for 35 min. As a result, mononuclear cells (MNC) collected on the surface of the Ficoll solution. The mononuclear cell layer or interphase layer was first drawn out and washed twice with a phosphate buffer before being spun in a centrifuge at 200×*g* for 10 min. The BM-MSCs were then grown and multiplied in a 25-ml culture flask using Roswell Park Memorial Institute (RPMI)-1640 medium for a period of 12 to 14 days at 37 °C with 5% CO_2_. The culture medium was refreshed every 2 to 3 days [13].

##### Utilizing flow cytometry to characterize MSC

Immunophenotyping was conducted by flow cytometry with immunostaining by monoclonal antibodies against CD 45 as a negative marker and CD 105 as a positive marker for MSC. Flow cytometry was used to assess the immune profile of MSCs, using the standard for MSC as described by the International Society for Cellular Therapy (ISCT). Cells (P2-3) were pelleted and resuspended in 1% bovine serum albumin in in phosphate-buffered saline (BSA in PBS) and counted. Each population containing 10^5^ cells was used for flow cytometry. Cells were stained directly using a concentration of 1:200 of phycoerythrin (PE)-conjugated CD105 antibodies (eBioscience, Germany) and CD 45 antibodies (eBioscience, Germany), and cells were incubated for 1 h. An appropriate isotype-matched control antibody named mouse immunoglobulin G1 (IgG1) K Iso control (eBioscience, Germany) was used in all analyses. The evaluation of the cells was performed using Cell Quest Software (Becton Dickinson, UK) on FACS flow cytometry [14].

#### Preparation, characterization, and quantification of exosomes from BM-MSCs

- * Preparation of exosomes derived from BM-MSCs*
  Exosomes were obtained from the supernatant of MSCs grown in RPMI media without the addition of fetal bovine serum (FBS). After removing any debris through differential centrifugation, the cell-free supernatant was further processed by being centrifuged for 1 h at 4 °C at 100,000×*g* using a Beckman Coulter Optima L-90 K ultracentrifuge. The exosomes underwent another round of ultracentrifugation after being washed with 25 mM solution of serum-free media 199 that contained *N*-2-hydroxy ethyl piperazine-*N*′-2-ethane sulfonic acid (from Sigma). The conditions were the same as before [10].
- *Identification of exosome derived from BM-MSCs*

##### Transmission electron microscopy (TEM)

The exosome was treated by washing it and then preserving it with 2.5% glutaraldehyde for 2 h. Afterward, it underwent ultracentrifugation and was mixed with 100 μL of human serum albumin (HSA). The exosome was mounted on a formvar-/carbon-coated grid and stained with 3% aqueous phosphor-tungstic acid in a negative manner. The morphology and dimensions of the separated exosome were visualized using transmission electron microscopy (TEM) on a Hitachi H-7650 microscope located in Japan [15].

##### Western blot analysis of CD81 and CD63

CD81 and CD63 are found in much higher quantities in exosomes compared to MSCs [16]. The first step in the process was to dissolve the exosome in a non-destructive lysis solution that contained a mixture of Tris HCL, NaCl, glycerol, Nonidet P-40, EDTA, and inhibitors for proteases and phosphatases. The solution was then exposed to sonication while being kept on ice, and the resulting fluid was collected by centrifuging it at 14,000×g for 30 min at a temperature of 4 °C. The solution was further examined using sodium dodecyl sulfate–polyacrylamide gel electrophoresis and transferred to nitrocellulose membranes for immunoblotting with targeted antibodies. The presence of immunoreactive bands was identified using a chemiluminescent substrate and horseradish peroxidase-conjugated secondary antibodies (Pierce, Rockford, IL).

The Alpha-Imager imaging equipment was used to scan and quantify the bands, while the Odyssey scanner from LI-COR was used to view and analyze the bands that were identified using IRDye-labeled antibodies [16].

(c) *Quantification of exosomes* produced from BM-MSCs by using shape and transmission electron microscopy methods.
(d) *Storage of ready-to-use exosome*

The exosome pellets were mixed with 100 µL of sterile water to create a diluted solution, which was then stored at – 80 °C until the designated time for injection.

#### Tissue sampling

Nine weeks later from the starting point of the experiment (1 week after stopping the treatment of groups III and IV by l-Dopa and exosomes), all animals were rendered unconscious with ether and then killed at the appropriate moment. Specimens from the brain tissue were delicately dissected, weighed, and cleaned three times with ice-cold saline to eliminate any unnecessary substances. Each specimen was then placed on ash-free filter paper and blotted. The brain was cooled on ice, and lower brain stem was taken and separated into two parts. The first part of the lower brain stem tissue was preserved in formalin (10%) and processed to create paraffin sections. These sections were then stained with hematoxylin and eosin (H&E), and photomicrographs were taken at varying magnifications for histopathological examination. Other sections were analyzed with immunohistochemistry to detect the tyrosine hydroxylase enzyme (TH) in all groups. The second part of the brain tissue was wrapped in aluminum foil and stored at − 70 °C until it was ready for analysis. The total protein content was calculated using the Bradford method [17].

#### Tyrosine hydroxylase (TH) immunohistochemistry

The specimens were prepared for paraffin sectioning by boiling tissue slices in sodium citrate solution (0.01 mol/L, pH 6) to reveal epitopes. These slices were then incubated overnight at 4 °C with primary anti-tyrosine hydroxylase polyclonal antibody (TH) from Abclonal (Woburn, MA, USA) in a 1:100 ratio. After the incubation, two washes with phosphate buffer were performed for 10 min each. The sections were then treated with a biotin-conjugated IgG in phosphate buffer for 1 h, followed by a 45-min application of premade avidin–biotin complex with peroxidase from Vector Laboratories. The bound complexes were then visualized using a 0.05% 3–3′-diaminobenzidine solution and a Mayer’s hematoxylin counterstain for 2 to 5 min [18].

##### Morphometric analysis

Calculate and find the average amount of TH-positive cells located in the SNc through immunostained sections. The interior of the TH+ cells had a brown appearance, whereas the cells without TH expression were either purple or blue. The cells were evaluated in 10 different areas of the slide at a ×400 magnification through the use of the ImageJ software program for each group.

#### Biochemical assessment

The levels of alpha-synuclein (α-SYN), DJ-1, and PARKIN in brain tissue were determined through the use of ELISA. The measurement of α-SYN was conducted with an ELISA kit (#AS-55550-R) purchased from AnaSpec in Fremont, California, USA [19]. The determination of DJ-1 was performed with an ELISA kit (#OKCA01920) obtained from Aviva Systems Biology Corp. in the USA [20]. The assessment of PARKIN was done utilizing an ELISA kit (#MBS722554) procured from My Biosource in San Diego, California, USA [21].

#### Genetic assessment

RNA extraction was carried out using the microRNANeasy Mini Kit (Catalog Number# 217004) obtained from QIAGEN in the USA [22]. The level of purification of the RNA concentrate was determined by determining its OD260 and OD260/280 ratio. To eliminate DNA contamination, the RNA was subjected to DNase I treatment.

##### Real-time PCR (qRT-PCR) for quantification of cirRNA 2837 and microRNA-34b

- The TransScript® Green One-Step qRT-PCR SuperMix kit (Catalog Number# AQ211-01) from Transgen Biotech [6] was utilized to measure the genetic expression of circRNA 2837. The expression was quantified relative to the β-actin housekeeping gene which was used as an internal control.
- The TransScript® Green microRNA Two-Step qRT-PCR SuperMix kit (Catalog Number# AQ202-01) from Transgen Biotech [23] was utilized to reverse transcribe targeted microRNAs and conduct quantitative RT-PCR for the expression of microRNA-34b. The expression was quantified relative to the RN U6B housekeeping gene.
- Sequence-specific primers were designed as follows:

**Table:** 

Gene	Primer sequence from 5′–3′ (F: Forward primer, R: Reverse primer)
circRNA 2837	(F), 5′-GATCCCAGCTCTTTCACCCG-3′; (R), 5′-CAACCAGCUAAGACACUGCGAAA-3′
microRNA-34b	(F), 5′-CGGCTCCCGGCCTGGGA -3′; (R), 5′-ACACCCCCGGGCCCAGC-3′
β-Actin	F: 5′-GGC GGCACCACCATGTACCCT-3 R: 5′-AGG GGCCGGACTCGTCATACT-3′
RN U6B	F: 5′-GCTTCGGCAGCACATATACTAAAAT-3′ R: 5′-CGCTTCACGAATTTGCGTGTCAT-3′

d. In order to perform the melting curve analysis, the temperature was increased from 63 to 95 °C during the final cycle. The cycle threshold (Ct) of the target genes was normalized with the help of the HKG using the StepOnePlus Real-Time thermal cycler and its specialized software. The relative genetic expression was then calculated using the Livak method and the 2^−ΔΔCt^ approach [24].

### Statistical analysis

The collected data in this study underwent a statistical analysis, and the outcome was presented in the form of mean ± SD through the use of SPSS version 23.0 for Windows. The significance of differences between the experimental groups was established through one-way ANOVA and Tukey’s tests. Pearson’s test was utilized to assess correlations, and a significance level of *P* < 0.05 was established.

## Results

### Behavioral testing results

**Fig. 2:**
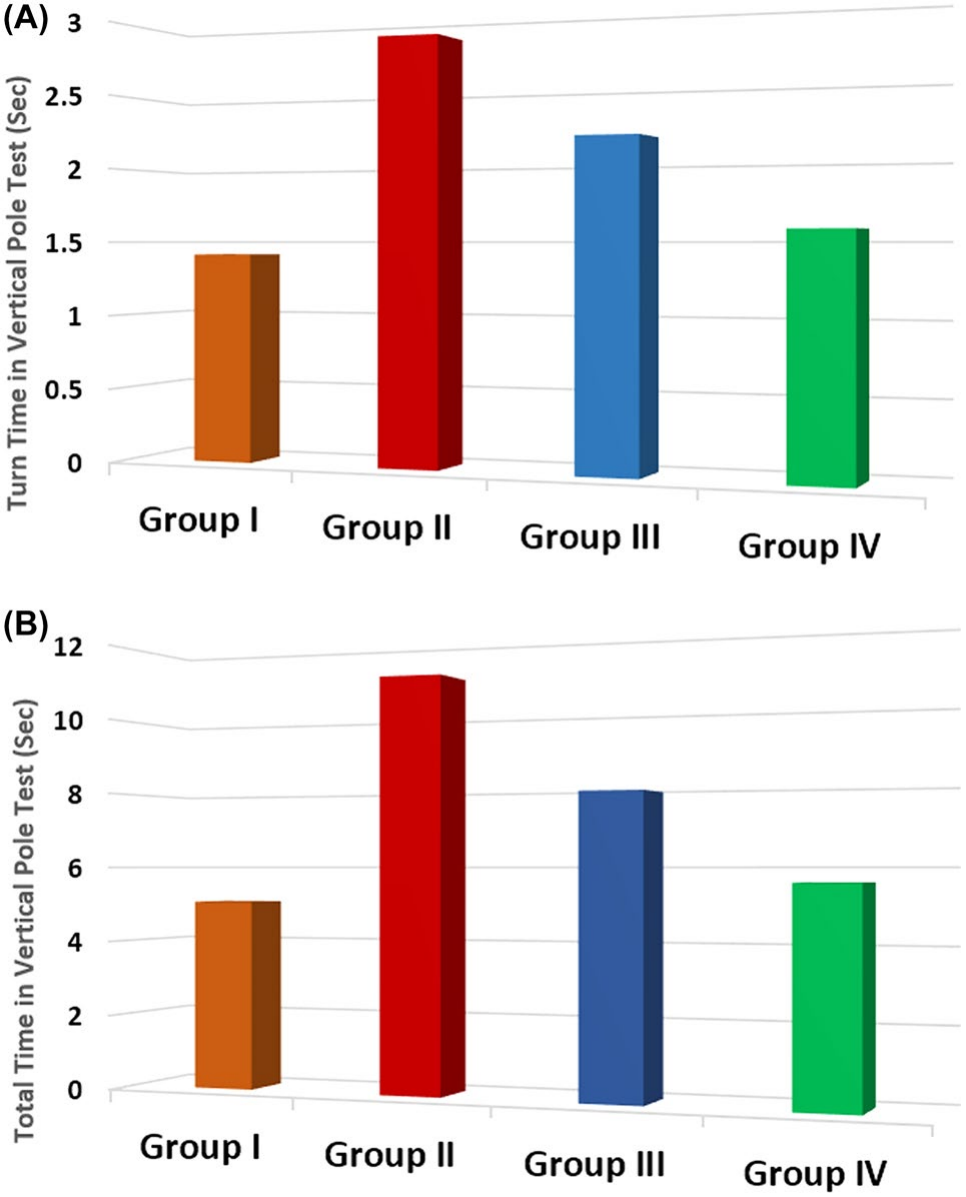
Comparison regarding turn time (**A**) and total time (**B**) (Sec) of vertical pole test in different studied groups along the course of the study

Administration of rotenone resulted in a progressive decline in movement speed, as demonstrated by the prolonged turn and total times in the pole test (*P* < 0.001). These deficits improved following treatment with exosomes and levodopa (*P* < 0.001) (Fig. 2A, B) (Table 1).

**Table 1:** Comparison of behavioral tests, tyrosine hydroxylase (TH)-positive cells, α-SYN, Parkin, DJ-1, miRNA-34b, and circRNA 2837 expression among the studied groups

			Group				ANOVA/tukey’s test	
			Group I (control group) (*n *= 10)	Group II (PD group) (*n* = 10)	Group III (PD-levodopa group) (n = 10)	Group IV (PD-MSCs-derived exosomes group) (*n* = 10)	*F*	*P*
Vertical Pole Test (Sec)	Turn Time		1.415 ± 0.293^b,c^	2.865 ± 0.490^a,c,d^	2.185 ± 0.252^a,b,d^	1.585 ± 0.162^b,c^	41.558	< 0.001*
	Total Time		5.065 ± 0.110^b,c^	11.026 ± 2.786^a,c,d^	7.950 ± 1.615^a,b,d^	5.624 ± 0.391^b,c^	27.913	< 0.001*
MWM Test (Sec)		Escape latency time	2.835 ± 0.226^b,c^	5.505 ± 0.259^a,c,d^	4.054 ± 0.408^a,b,d^	3.054 ± 0.134^b,c^	195.539	< 0.001*
		Retention time	16.935 ± 0.466^b,c^	10.995 ± 0.403^a,c,d^	13.235 ± 0.471^a,c,d^	17.055 ± 0.433^b,c^	444.808	< 0.001*
(TH)-positive cells			24.800 ± 3.393^a,b,c^	8.100 ± 4.012^a,c,d^	13.300 ± 3.802^a,b,d^	19.400 ± 3.688^a,b,c^	37.859	< 0.001*
α-SYN (pg/mg protein)			49.420 ± 9.406^b,c^	156.600 ± 14.334^a,c,d^	81.830 ± 20.456^a,b,d^	59.840 ± 11.043^b,c^	112.198	< 0.001*
Parkin (pg/mg protein)			11.790 ± 3.347^b,c^	6.440 ± 1.435^a,d^	7.740 ± 3.744^a^	10.230 ± 3.259^b^	6.131	0.002*
DJ-1 (ng/mg protein)			3.490 ± 0.441^a,b,c^	1.190 ± 0.269^a,c,d^	2.430 ± 0.236^a,c,d^	3.040 ± 0.341^a,b,c^	90.908	< 0.001*
miRNA-34b relative expression			1.031 ± 0.143^b,c^	0.263 ± 0.074^a,d^	0.351 ± 0.094^a,d^	0.914 ± 0.109^b,c^	130.404	< 0.001*
circRNA 2837 relative expression			1.256 ± 0.067^b,c^	1.778 ± 0.182^a,d^	1.710 ± 0.107^a,d^	1.152 ± 0.145^b,c^	56.796	< 0.001*

Data are mean + standard deviation (SD); *n* number of rats. Statistical analysis is carried out using one-way analysis of variance (ANOVA) followed by Tukey’s test. Lowercase letters a–d indicate significant differences between groups at *P* < 0.05: “a” denotes the significance from the control group (I); “b” denotes the significance from PD group (II); “c” denotes the significance from PD-levodopa group (III); “d” denotes the significance from PD-MSCs-derived exosomes group (IV)

*PD* Parkinson’s disease, *MWM* Morris water maze test, *TH* tyrosine hydroxylase, *α-SYN* α-synuclein, *DJ-1* protein deglycase, *miRNA-34b* microRNA-34b, *circRNA 2837* circularRNA2837

**Fig. 3:**
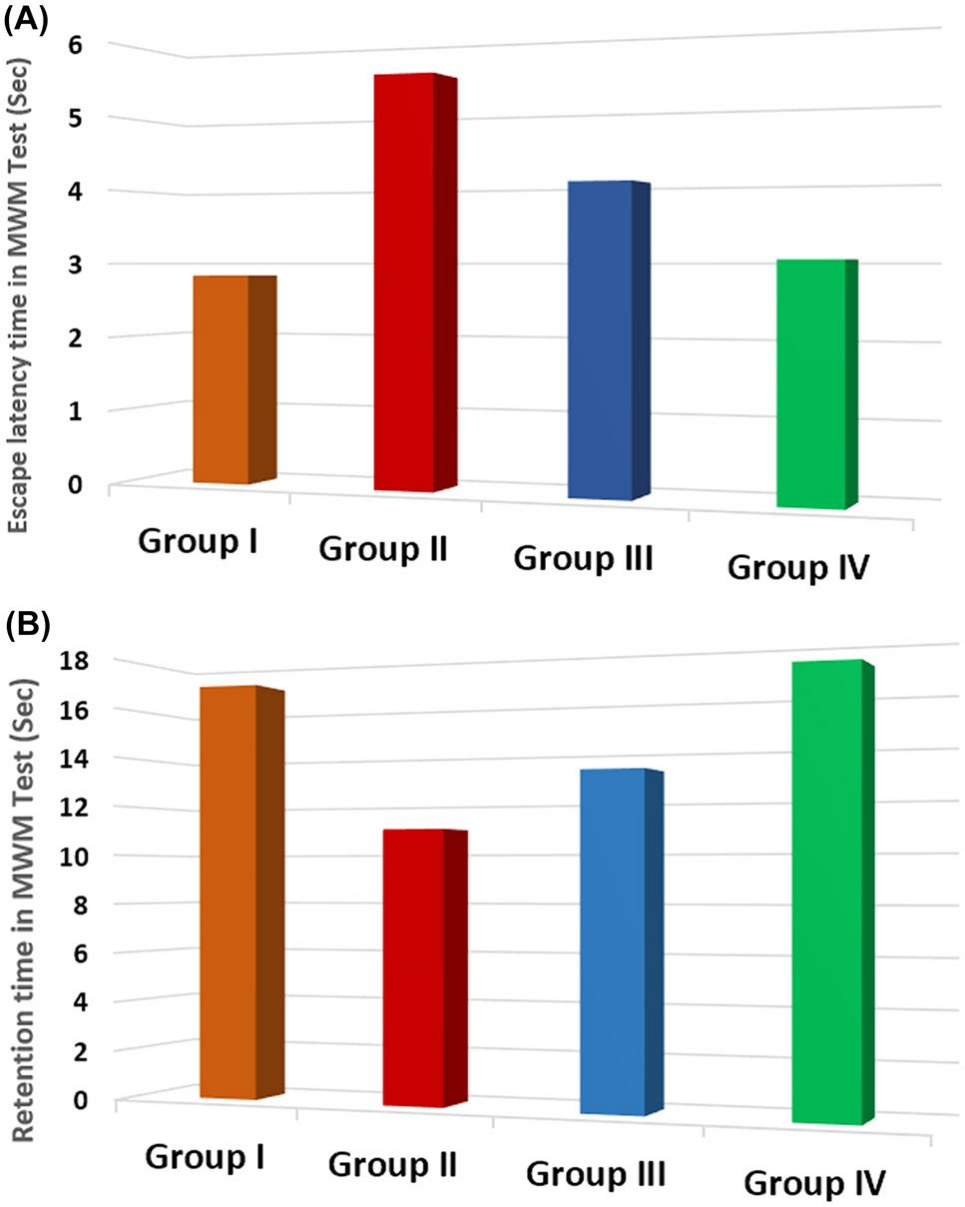
Comparison regarding escape latency time (**A**) and retention time (**B**) (Sec) of Morris water maze test in different studied groups along the course of the study

The administration of rotenone caused a decline in cognitive ability, as shown by a longer escape time in the Morris water maze test, compared to the control group (P < 0.001). However, this decline was reversed with exosomes and levodopa treatment (*P* < 0.001) (Fig. 3A). Additionally, the injection of rotenone significantly reduced the retention time (*P* < 0.05 compared to the control group), but this decrease was also improved with exosomes and levodopa treatment (*P* < 0.001) (Fig. 3B) (Table 1).

### Microscopic cell morphology of isolated BM-MSC

**Fig. 4:**
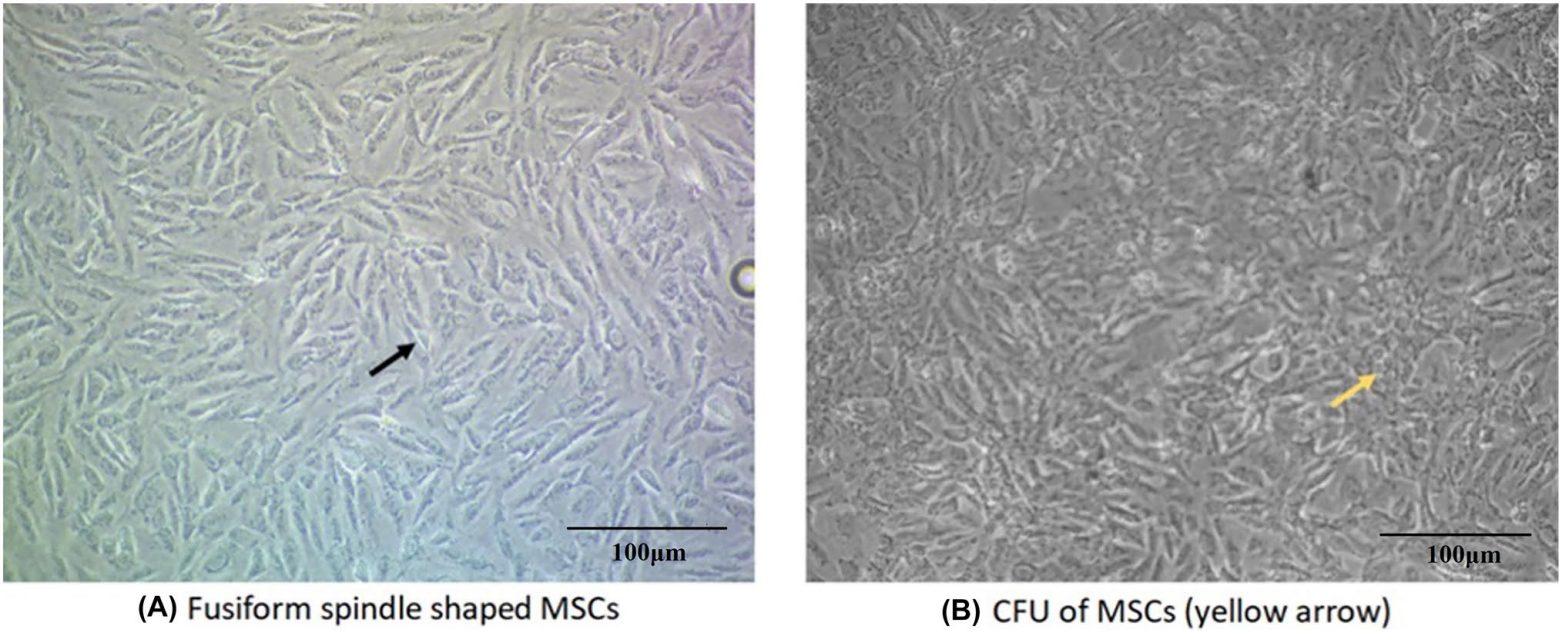
MSCs in culture. MSCs were identified through their morphology as they look spindle-shaped fibroblast-like cells, as well as their colony-forming unit (CFU). Scale bar = 100 μm

MSCs were identified by their morphological spindle-shaped fibroblast-like cells and colony-forming unit (CFU) (Fig. 4).

### Flow cytometric result of BM-MSC

**Fig. 5:**
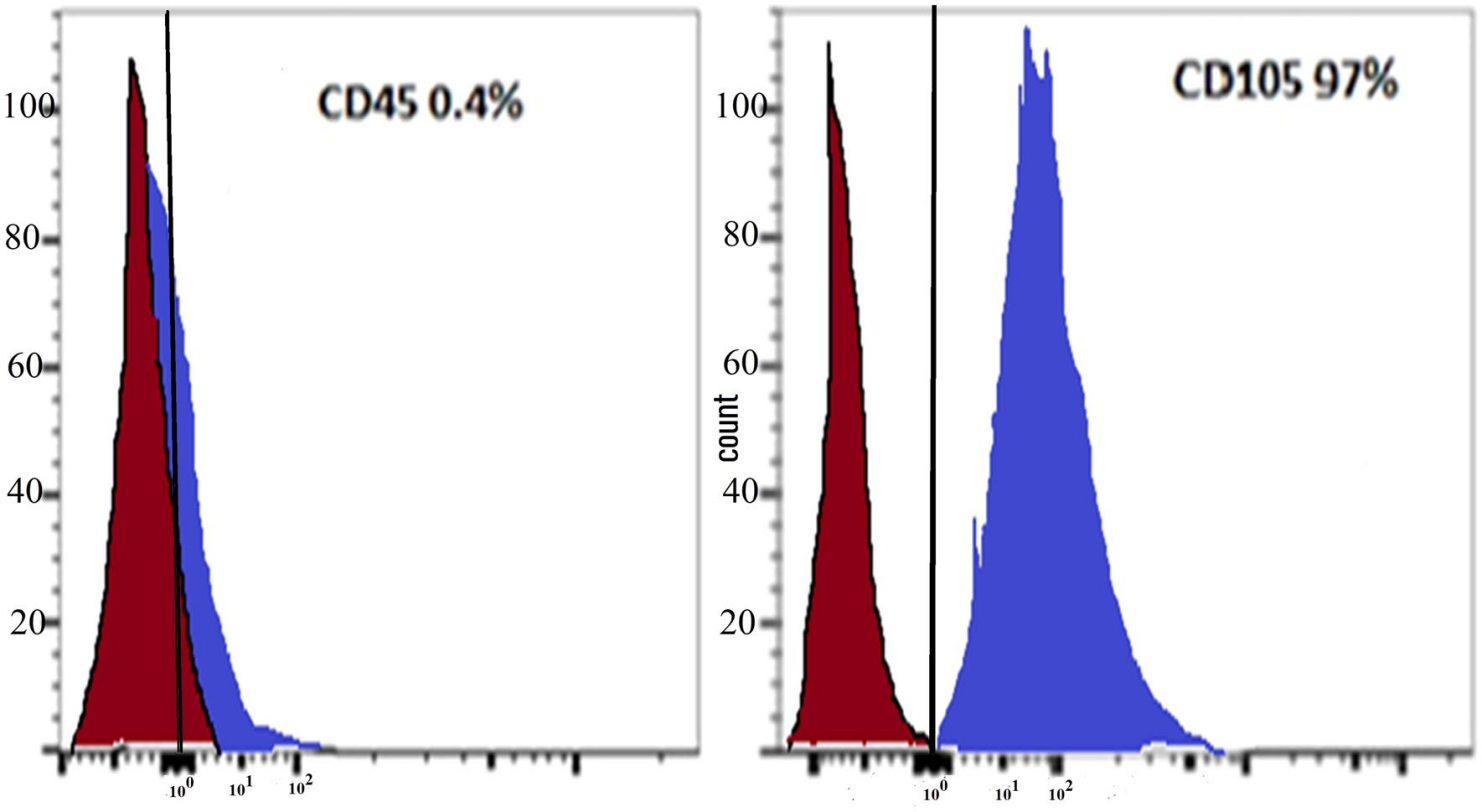
MSCs characterization by flow cytometry analysis. MSCs are negative for surface marker CD45 (0.4%), but positive for CD105 (97%)

BM-MSCs demonstrated a low expression of the surface marker CD45 (0.4%) and a high expression of CD105 (97%) (Fig. 5).

### Exosome characterization by TEM

**Fig. 6:**
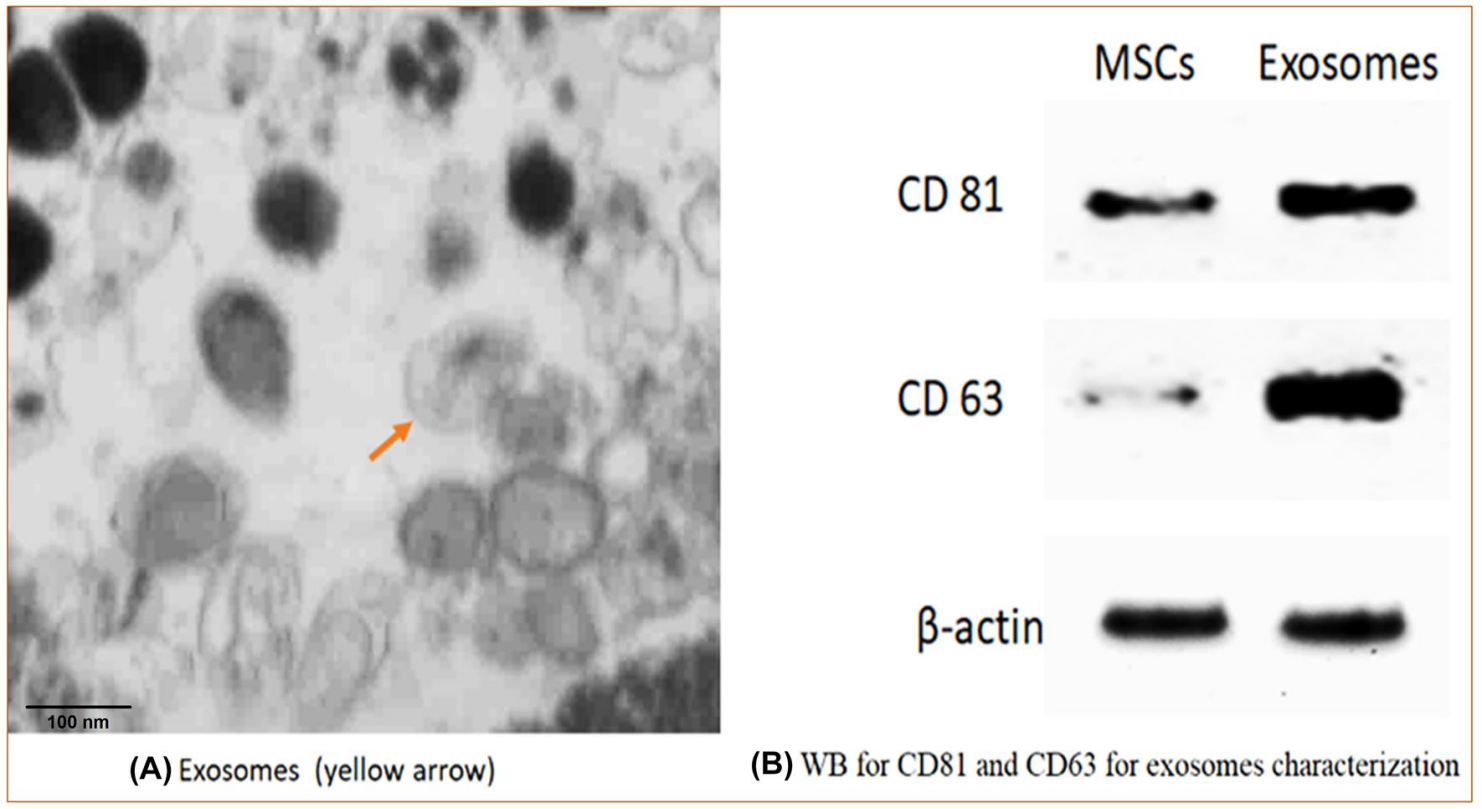
Exosome characterization by TEM and Western Blotting. **A** Exosomes were characterized through TEM by their cup-shaped spheroidal morphology and their size was about 100 nm, scale bar = 100 nm. **B** Western blotting analysis shows a positive result for surface marker CD81 and CD63, which are characteristics for exosomes [(Note: β-actin in **B**) is the reference protein (internal control)]

The exosome derived from BM-MSCs was characterized through TEM and found to have a cup-shaped spheroidal morphology, with an average size of 30–100 nm (Fig. 6A).

### Exosome characterization by Western blot

WB analysis shows a positive result for surface markers CD81 and CD63, which are characteristic of exosomes (Fig. 6B).

### Histopathological results: (Fig. 7)

#### Group I (control)

**Fig. 7:**
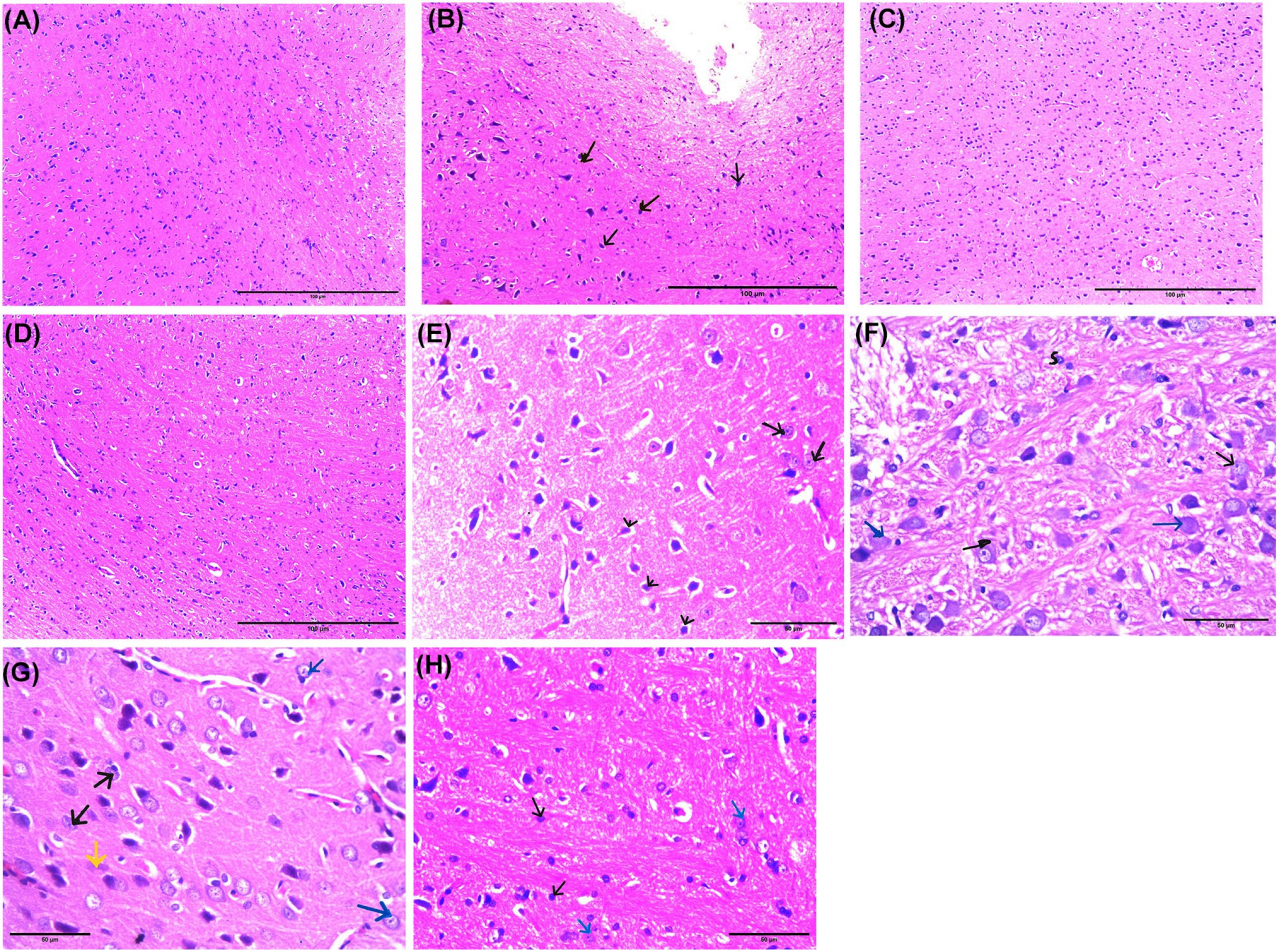
Histopathological sections in the SN of different studied groups (H&E). **A**–**D** H&E × 100, scale bar = 100 μm]. **A** Section in the SN of group (I) showing normal neurons of variable sizes and shapes. The glial cells are smaller and darker than the neurons and have dense nuclei. **B** Section in the SN of group (II) showing neuronal loss with increased glial cells (arrows). **C** Section in the SN of group (III) showing neurons, glial cells, and dilated blood vessels. **D** Section in the SN of group (IV) showing neurons of different shape and size and glial cells. **E–H** H&E × 400, scale bar = 50 μm]. **E** section in the SN of group (I) showing neurons with large pale nuclei, prominent nucleoli and lightly stained cytoplasm (black arrow). The glial cells are smaller and darker have dense nuclei. **F** section in the SN of group (II) showing neurons having nuclei with peripheral condensation of the chromatin (black arrow). Some of them have typical hyaline cytoplasmic inclusions—Lewy bodies (blue arrow). The neurons are surrounded by perineuronal spaces. Other deeply stained shrunken neurons (S) are observed. **G** section in the SN of group (III) showing some neurons having rounded nuclei with nucleoli (blue arrow). Other neurons (black arrow) have nuclei with peripheral condensation of the chromatin, while Lewy body is seen in some neurons (yellow arrow). **H** section in the SN of group (IV) showing neurons having large pale nuclei, prominent nucleoli, and lightly stained cytoplasm (black arrow) admixed with smaller glial cells

Light microscopic (L/M) examination of the section in the substantia nigra (SN) reveals neurons with large pale nuclei, prominent nucleoli, and lightly stained cytoplasm (as indicated by the black arrow). In contrast, the glial cells in the same section are smaller and darker, with dense nuclei (Fig. 7E).

#### Group II (PD group)

Section in the SN displays neurons with nuclei that exhibit peripheral chromatin condensation, indicated by the black arrow. Some neurons have distinctive hyaline cytoplasmic inclusions known as Lewy bodies, indicated by the blue arrow. These neurons are surrounded by perineuronal spaces. Additionally, the section displays deeply stained, shrunken neurons (S) (Fig. 7F).

#### Group III (PD-l-Dopa group)

Section in the SN of group III showing some neurons with rounded nuclei and nucleoli (blue arrow). Other neurons (black arrow) display peripheral chromatin condensation in their nuclei, while some neurons exhibit Lewy bodies (yellow arrow) (Fig. 7G).

#### Group IV (PD-BM-MSC-derived exosome group)

Section in the SN of group IV demonstrates neurons with large, pale nuclei, prominent nucleoli, and lightly stained cytoplasm (blue arrow) mixed with smaller glial cells (Fig. 7H).

### Immunohistochemical results: (Fig. 8)

#### Group I (control)

**Fig. 8:**
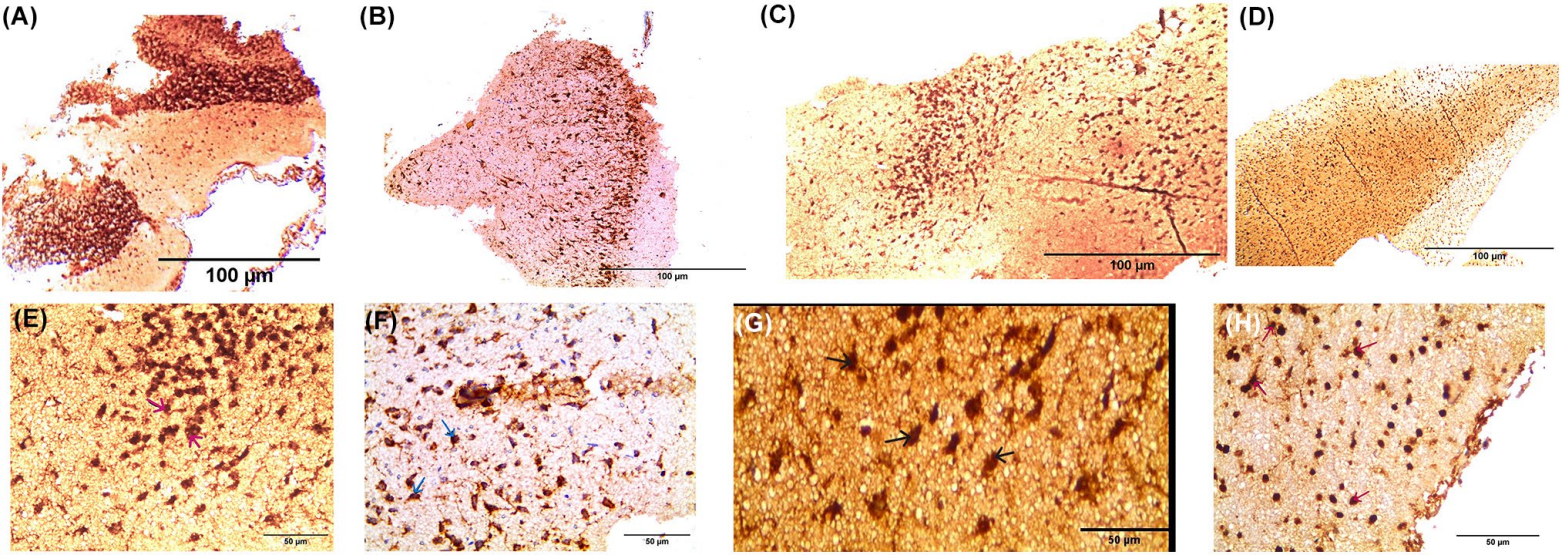
Tyrosine hydroxylase (TH)-immunostained sections in different studied groups. **A–D** IHC × 100, scale bar = 100 μm]. A TH-immunostained section in the SN of group (I) showing strong positive TH immunoreactivity in neurons. B TH-immunostained section in the SN of group (II) showing few mild positive neurons. C TH-immunostained section in the SN of group (III) showing moderate TH immunoreactivity of the neurons. D TH-immunostained section in the SN of group (VI) showing strong positive TH-stained neurons. **E–H** IHC × 400, scale bar = 50 μm]. E TH-immunostained section in the SN of group (I) showing strong positive TH-stained neurons (arrows) and the surrounding dense TH-immunoreactive neuropil. F TH-immunostained section in the SN of group (II) showing few mild positive TH-stained neurons (arrows) and weak positivity of the neuropil. G TH-immunostained section in the SN of group (III) showing moderate TH immunoreactivity of the neurons. H TH-immunostained section in the SN of group (VI) showing strong positive TH-stained neurons (arrows) and the surrounding neuropil

The TH-immunostained section in the SN of group I displays strong positive TH-stained neurons (as indicated by arrows) and the surrounding dense TH-immunoreactive neuropil, viewed at a higher magnification of IHC × 400 (Fig. 8A, E).

#### Group II (PD group)

In the TH-immunostained section of group (II) (Fig. 8B, F), there are few mild TH-positive neurons indicated by arrows, and the neuropil shows weak positivity.

#### Group III (PD-l-Dopa group)

TH-immunostained section in the SN of group III reveals moderate TH immunoreactivity in the neurons (arrows) (Fig. 8C, G).

#### Group IV (PD-BM-MSC-derived exosome group)

A TH-immunostained section in the SN of group VI displays strong positive TH-stained neurons (arrows) and the accompanying neuropil (Fig. 8D, H).

### Morphometric results

**Fig. 9:**
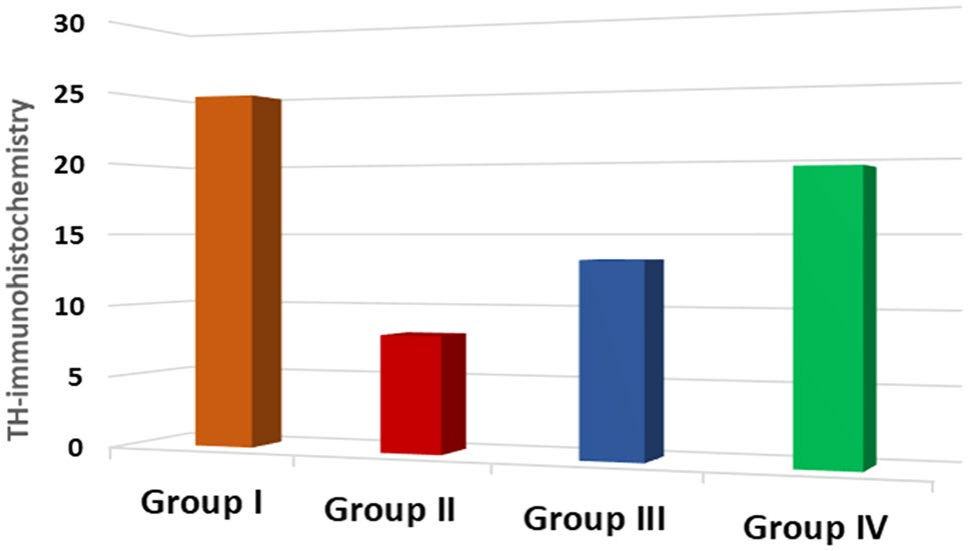
Comparison regarding TH-positive cells in the SN among the studied groups

In each group, the number and quantity of TH+ DAn in the SNc were counted. A substantial reduction was discovered when comparing the PD group to the controls. Treatment with exosomes derived from PD-BM-MSC (PD-BM-MSC-derived exosome group) and PD therapy with l-Dopa (PD-l-Dopa group) enhanced the number of TH+ DAn compared to the PD group. There was a statistically significant difference between the PD-BM-MSC-derived exosome group (IV) and the PD-l-Dopa group (III) (Fig. 9) (Table 1).

### Biochemical results

**Fig. 10:**
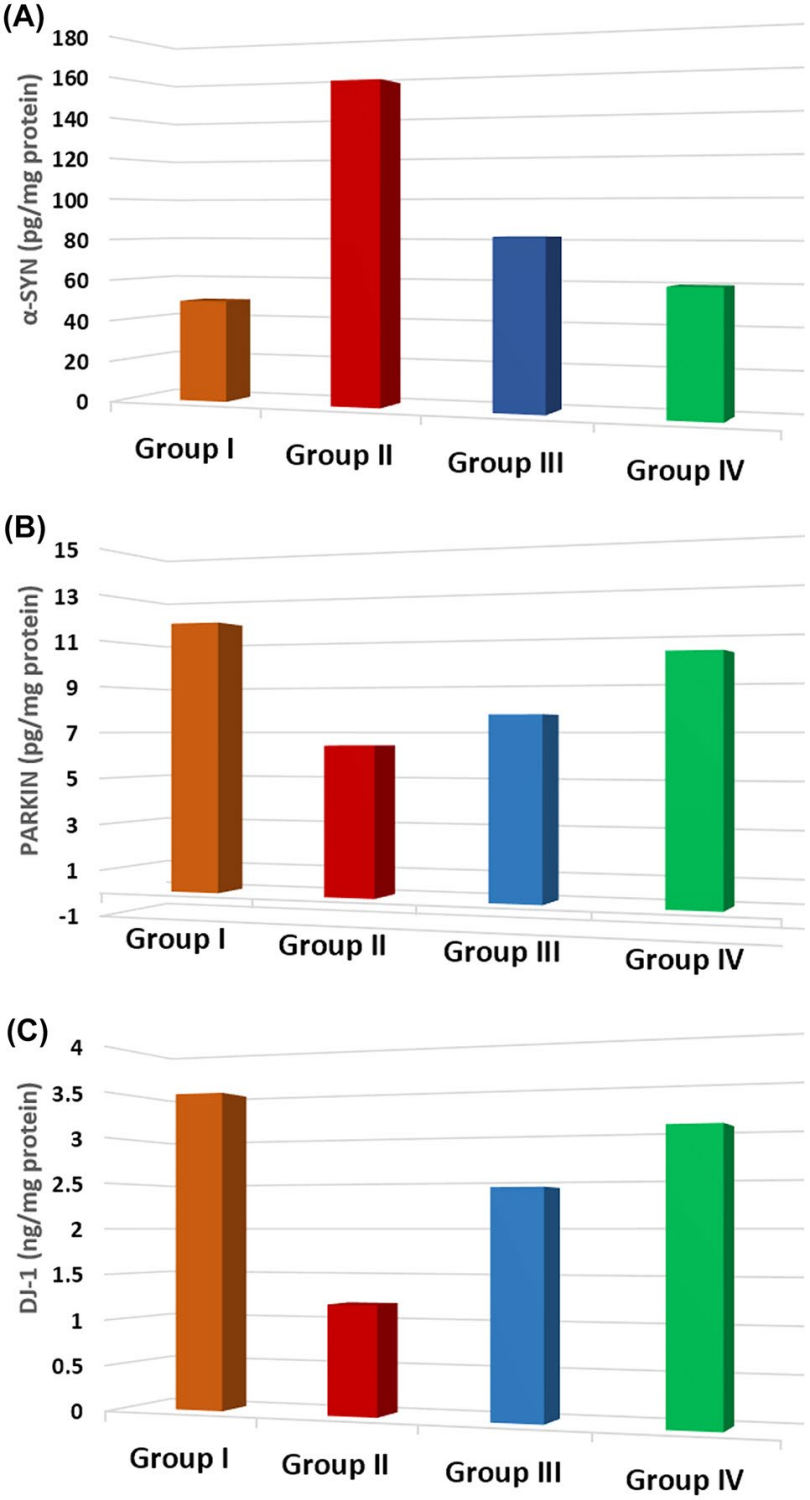
Comparison regarding brain tissue α-SYN, Parkin, and DJ-1 levels among the studied groups

The levels of α-SYN showed a statistically significant increase in the PD group compared to the controls, with a *P* value less than 0.05. However, the levels of α-SYN decreased in the PD-l-Dopa group and the PD-MSCs-derived exosome group, compared to the PD group, with a *P* value less than 0.05. Additionally, there was a statistically significant difference between the PD-MSCs-derived exosome group (IV) and the PD-l-Dopa group (III), with a *P* value less than 0.05 (Fig. 10A) (Table 1).

Brain tissue levels of parkin and DJ-1 were significantly lower in the PD group (II) compared to the control group (*P* < 0.05), but were significantly higher in the PD-l-Dopa group (III) and the PD-MSCs-derived exosome group (IV) compared to the PD group (*P* < 0.05). This is shown in Fig. 10B and C and Table 1.

### Genetic results

**Fig. 11:**
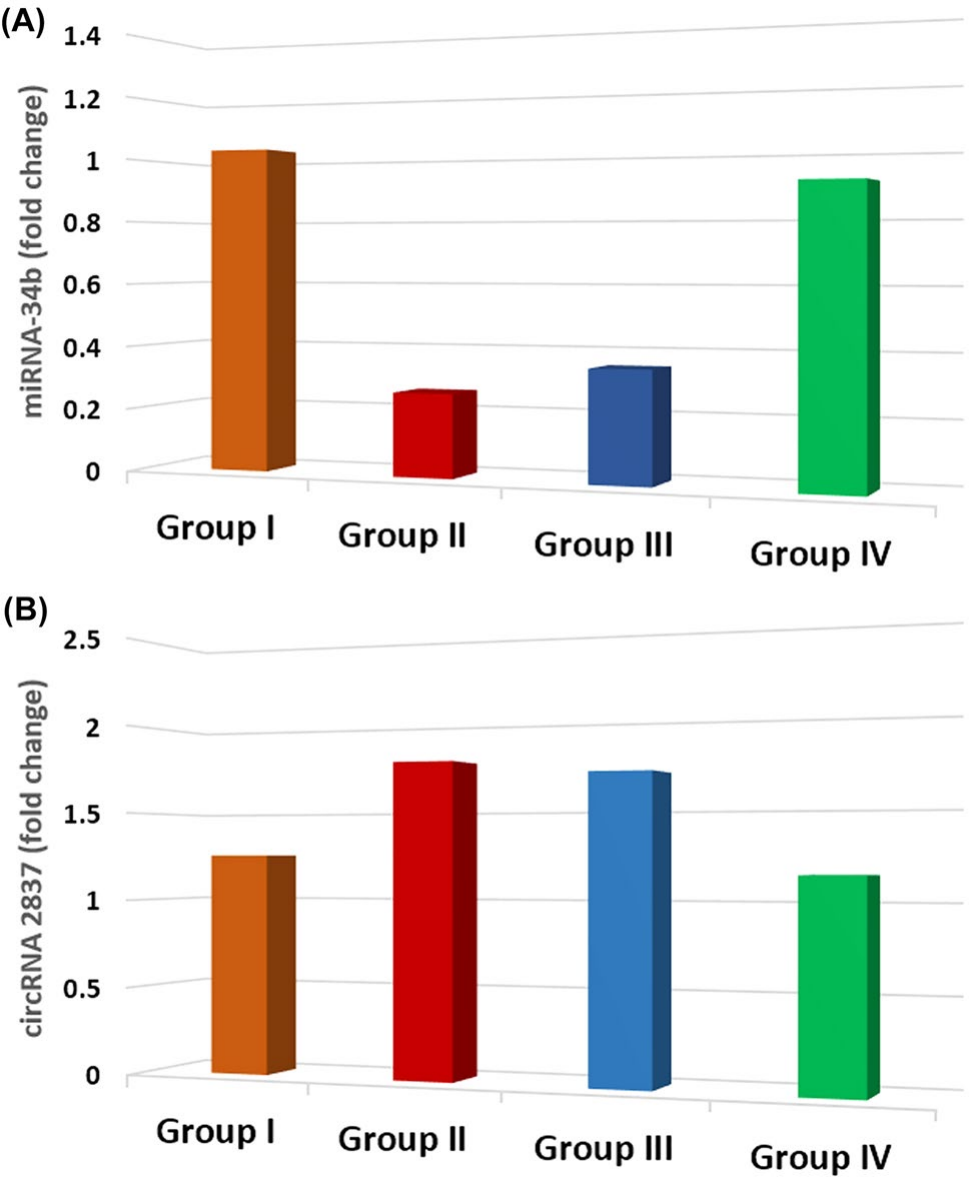
Comparison regarding brain tissue miRNA-34b and circRNA 2837 expression levels (fold change) among the studied groups

The PD group demonstrated a reduction in the amount of microRNA-34b compared to the control group, and this decrease was statistically significant (*P* < 0.05). There was no notable variation in the expression of microRNA-34b between the PD and PD-l-Dopa groups (*P* = 0.282). However, the PD-MSCs-derived exosome group showed a statistically significant increase in the expression of microRNA-34b when compared to both the PD group and PD-l-Dopa group (*P* < 0.05) (Fig. 11A) (Table 1).

The PD group showed a statistically significant increase in circRNA 2837 expression compared to the controls (*P* < 0.05). There was no significant difference in the values of circRNA 2837 expression between the PD and PD-l-Dopa groups (III) (*P* = 0.662). Furthermore, the PD-MSCs-derived exosome group (IV) showed a statistically significant decrease in circRNA 2837 expression compared to the PD and PD-l-Dopa groups (III) (*P* < 0.05) (Fig. 11B) (Table 1).

### Correlation matrix for the studied biochemical and molecular parameters

**Fig. 12:**
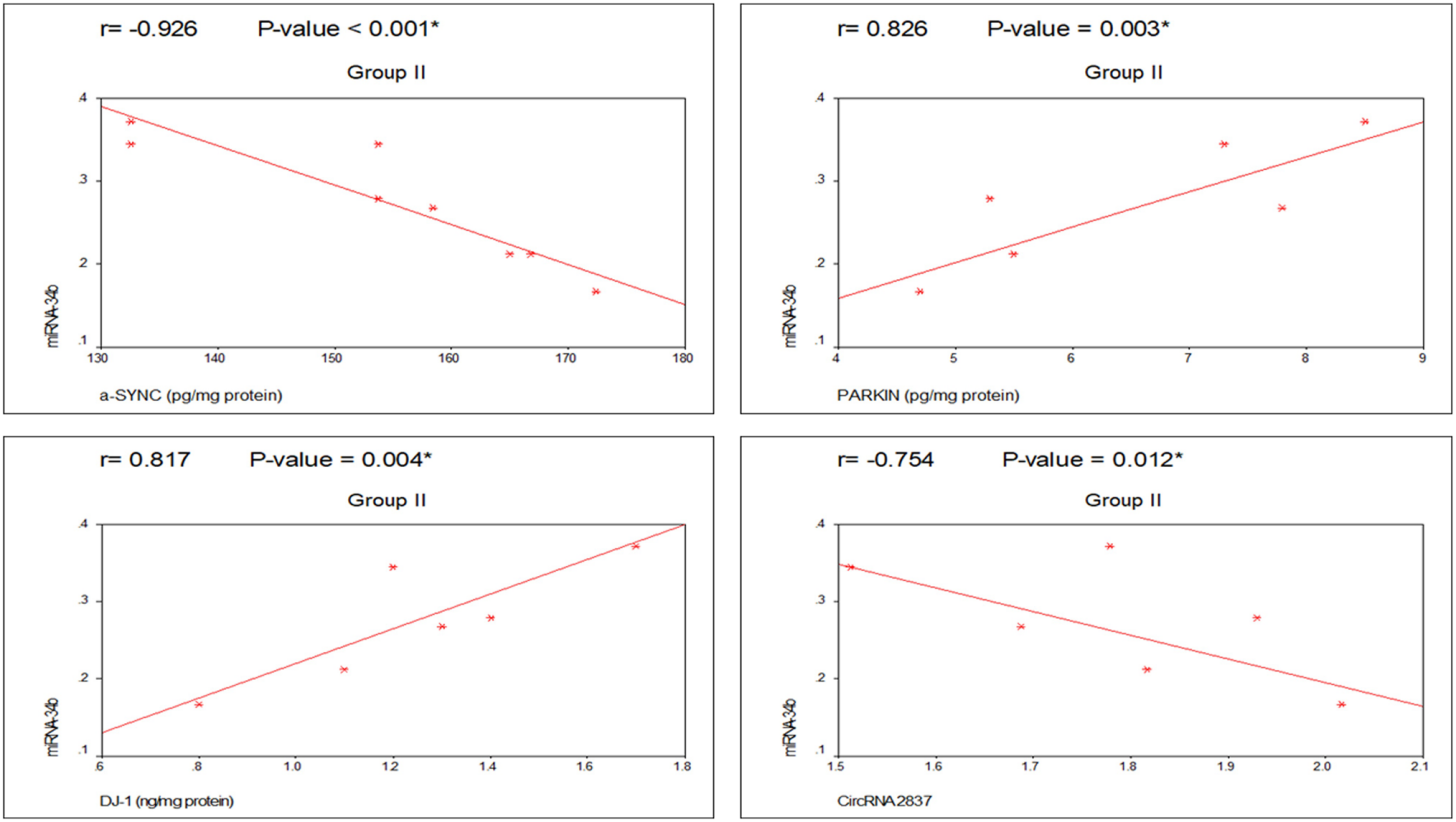
Correlation between the studied parameters in group II (PD group)

**Fig. 13:**
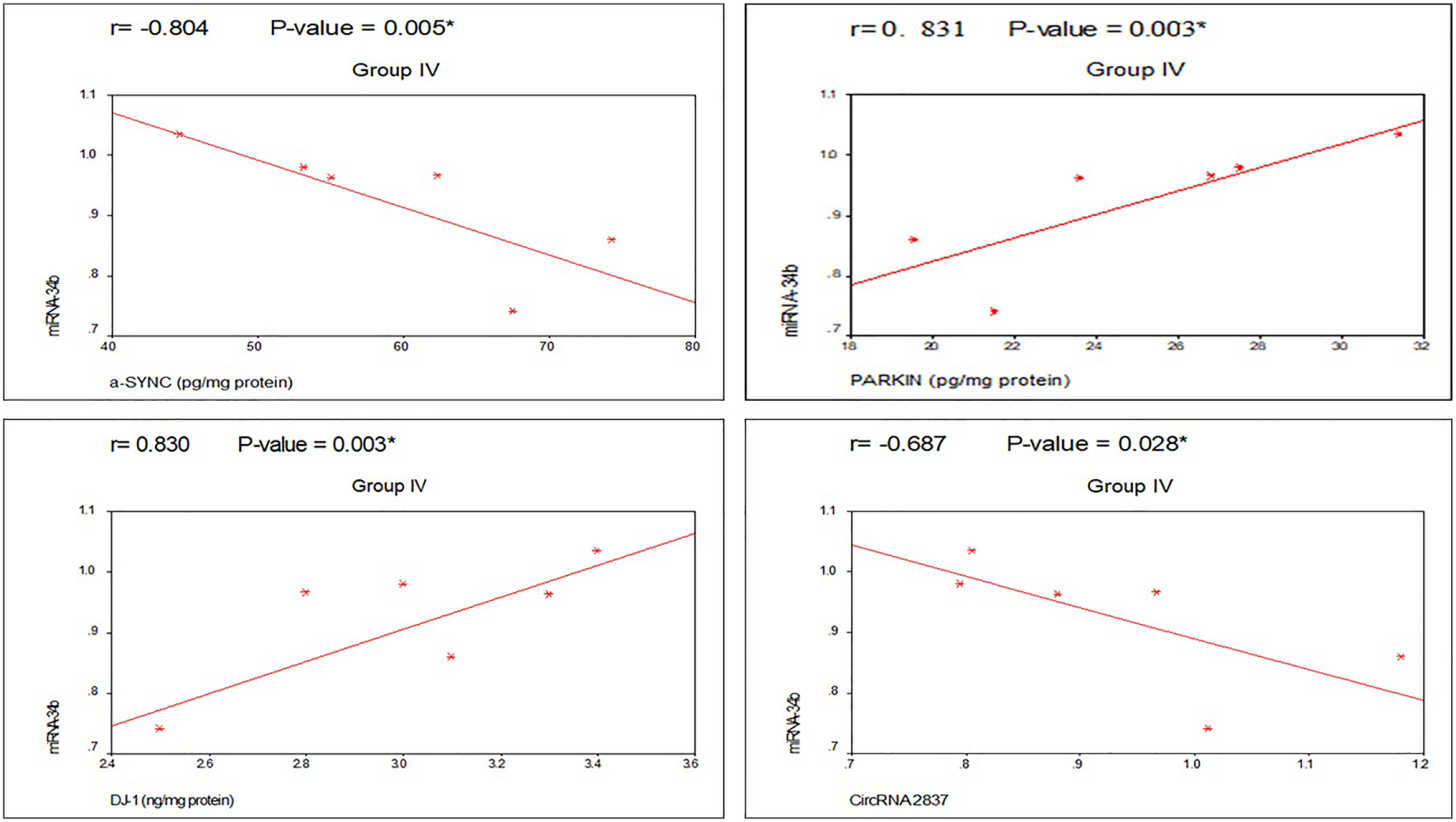
Correlation between the studied parameters in group IV (PD-MSCs-derived exosomes group)

The correlations between the PD group (II) and the PD-MSCs-derived exosome group (IV) are summarized in Tables 2 and 3. They showed statistically significant negative correlations with microRNA-34b and α-SYN. However, microRNA-34b was significantly positively correlated with PARKIN and DJ-1. Additionally, there was a statistically significant negative correlation between brain tissue microRNA-34b and circRNA 2837, as shown in Figs. 12 and 13.

**Table 2:** Correlation between α-SYN, Parkin, DJ-1, miRNA-34b, and circRNA 2837 expression in group II (PD group)

Correlations					
Group II (PD group)		α-SYN (pg/mg protein)	PARKIN (pg/mg protein)	DJ-1 (ng/mg protein)	CircRNA2837 (fold change)
PARKIN (pg/mg protein)	*r*	− 0.739			
	*P* value	0.015*			
DJ-1 (ng/mg protein)	*R*	− 0.771	0.765		
	*P* value	0.009*	0.010*		
CircRNA2837 (fold change)	*R*	0.623	− 0.756	− 0.423	
	*P* value	0.055	0.011*	0.223	
miRNA-34b (fold change)	*R*	− 0.926	0.826	0.817	− 0.754
	*P* value	< 0.001*	0.003*	0.004*	0.012*

*PD* Parkinson’s disease, *α-SYN* α-synuclein, *DJ-1* protein deglycase, *miRNA-34b* microRNA-34b, *circRNA 2837* circularRNA2837

*Significant *P* value (< 0.05)

**Table 3:** Correlation between α-SYN, Parkin, DJ-1, miRNA-34b, and circRNA 2837 expression in group IV (PD-MSCs-derived exosomes group)

Correlations					
Group IV (PD-MSCs-derived exosomes group)		α-SYN (pg/mg protein)	PARKIN (pg/mg protein)	DJ-1 (ng/mg protein)	CircRNA 2837 (fold change)
PARKIN (pg/mg protein)	*r*	− 0.882			
	*P* value	0.001*			
DJ-1 (ng/mg protein)	*r*	− 0.602	0.443		
	*P* value	0.066	0.200		
CircRNA 2837 (fold change)	*r*	0.954	− 0.855	− 0.397	
	*P* value	< 0.001*	0.002*	0.256	
miRNA-34b (fold change)	*r*	− 0.804	0.831	0.830	− 0.687
	*P* value	0.005*	0.003*	0.003*	0.028*

*PD* Parkinson’s disease, *α-SYN* α-synuclein, *DJ-1* protein deglycase, *miRNA-34b* microRNA-34b, *circRNA 2837* circularRNA2837

*Significant *P* value (< 0.05)

## Discussion

Although 10–15% of Parkinson’s disease (PD) cases are hereditary [25], the majority of cases are idiopathic. Environmental factors, such as neurotoxins, are critical in the etiology of PD. Chronic subcutaneous exposure to rotenone leads to replication of PD traits through free radical overproduction driven by oxidative stress, resulting in preferential degeneration of dopaminergic neurons (DAn) [26].

In this study, 2 mg/kg/day of rotenone was administered subcutaneously for 5 weeks to induce Parkinson’s disease (group II) [7]. The effectiveness of this treatment was confirmed through neurobehavioral, learning, and spatial memory assessments, as well as histopathological studies of brain tissue.

Our data revealed a marked decline in neurobehavioral aspects, spatial memory, and learning that were assessed by the pole test and MWM test after rotenone injection in comparison to controls. On a histopathological level, degenerative changes in the SNc were found on examination of brain tissues from the PD group.

TH deficiency leads to impaired synthesis of dopamine and alterations in its activity, which are involved in disorders such as Parkinson’s disease (PD) and schizophrenia [27]. In the present study, rot-injected animals showed a statistically significant decline in TH immunoreactivity and obvious neuronal cell loss in the substantia nigra pars compacta (SNc). Morphometric analyses revealed decreased TH+ cells, which was statistically significant in the PD group (II) compared to the control group (I), indicating a reduction in TH levels in the PD group.

The way in which rotenone causes Parkinson’s disease involves disruptions in the mitochondrial electron transport chain (ETC), causing an excessive amount of reactive oxygen species (ROS), oxidative stress (OS), and resulting in the degeneration of dopamine-rich nuclei and the aggregation of alpha-synuclein [28].

DJ-1, an OS-induced chaperone, prevents α-syn fibrillation [29]. Confirmation was made that suppression of DJ-1 increased the level of aggregated α-syn in PD [30].

PARKIN, a cytosolic ubiquitin ligase, plays a crucial role in regulating mitochondrial quality control by eliminating damaged or dysfunctional mitochondria [31, 32]. The presence of malfunctioning mitochondria can lead to cell degeneration through a process called mitophagy in PD [33]. In this study, the group of rats treated with rotenone (group II) showed a statistically significant increase in α-syn levels and a statistically significant suppression of PARKIN and DJ-1 proteins compared to the control group (I).

MicroRNA-34b has been found to be downregulated in several brain areas in Parkinson’s disease (PD) with varying degrees of impact on key pathways in the disease’s pathogenesis [34]. This microRNA targets the 3′-UTR of α-syn mRNA, causing a decrease in α-syn protein levels. Inhibition of microRNA-34b has been shown to result in increased α-syn expression and the formation of α-syn-containing aggregates, known as Lewy bodies [2]. Our observations indicate that microRNA-34b depletion in the PD group (II) affects pathways that regulate PARKIN and DJ-1 levels, leading to a reduction in both. Deficiencies in either Parkin or DJ-1 cause mitochondrial dysfunction and oxidative damage.

In recent years, circRNA has become a popular area of research in the field of non-coding RNA (ncRNA). CircRNA has a number of functions, including the ability to absorb microRNA through its microRNA-binding sites, acting as a “sponge” and reducing the inhibition of microRNA on target genes [35]. This results in increased expression of target genes through a circRNA-microRNA-mRNA pathway. CircRNAs are also seen as potential markers for neurodegenerative illnesses and aging studies.

In this study, we analyzed the expression of circRNA.2837 in the brain tissues of a Parkinson’s disease (PD) group. Our qRT-PCR results showed that the expression of circRNA.2837 was significantly upregulated in PD mice (group II) compared to the control group (I). We also found that the expression of circRNA.2837 was negatively correlated with the expression of microRNA-34b, indicating a direct interaction between these RNAs.

So, for the previously stated reason, this study was conducted to clarify the role and differentiate the effectiveness of treatment with mesenchymal stem cell (MSC)-derived exosomes versus l-Dopa in inducing Parkinson’s disease (PD) in rats. The aim was to determine their potential for neurogenic repair and functional restoration.

In this study, group III, after PD induction, was given oral Sinemet® tablets (Sinemet®, 250/25 l-Dopa/carbidopa) crushed in water and administered through oral gavage, 12 mg/Kg twice daily for 3 weeks [8].

In the current study, rats that received l-Dopa (group III) showed increased levels of PARKIN and DJ-1 and a reduction in α-syn level compared to the PD group (II). There were no statistically significant differences in the expression levels of microRNA-34b and circRNA 2837 between group III and group II (*P* = 0.282 and *P* = 0.662, respectively). Although l-Dopa has antioxidant properties, its use may cause side effects and toxicity as demonstrated in vitro assays due to the generation of toxic ROS at higher concentrations that can harm neurons.

The effects of dopamine (DA) are dose dependent, with low to moderate amounts being neuroprotective, but greater concentrations being harmful. Currently, the only approved treatment for Parkinson’s disease (PD) is l-Dopa, despite its limitations, diverse side effects, and lack of efficacy for non-dopaminergic symptoms such as cognitive impairment. To date, no cure exists. As a result, among the unique treatment techniques, the use of stem cells has gained particular attention [36].

With the transportation of lipids, protein molecules, messenger RNA (mRNA), and microRNA, exosomes are now widely accepted to play a role in intercellular communication. These tiny particles, due to their unique features, can alter the physiological activity of many cells in the body. There is statistical evidence that exosomes play a role in tissue healing, stimulation, and modulation of immunity. Therefore, they can be tested for their potential to regenerate nerve cells in neurodegenerative disorders [37].

In this study, group IV rats were given an intravenous dose of BM-MSCs-derived exosome 1 ml (in the rat tail) once a week for three consecutive weeks [9].

In this study, rats in group IV who received exosomes showed a statistically significant increase in the levels of PARKIN and DJ-1, as well as a significant reduction in α-syn level compared to PD group II. There was also a statistically significant difference in the levels of DJ-1 and α-syn between group III and group IV. Furthermore, group IV showed a statistically significant increase in the expression of microRNA-34b and a statistically significant repression of circRNA.2837 compared to group III and group II.

The treated groups showed improvement in neurobehavioral tests, with a moderate improvement in group III and a marked improvement in group IV compared to PD group II. The histological results also showed that treatment with exosomes (group IV) and l-Dopa (group III) significantly increased TH+ DAn compared to PD group II. A statistically significant difference was found between group IV and group III, suggesting that exosomes had a regenerative effect.

These findings are supported by those reported by Meligy et al. who stated that mesenchymal stem cells (MSCs) producing exosomes have the potential to differentiate into neuron-like cells. Exosome transplantation in mice with Parkinson’s disease (PD) resulted in the recovery of dopamine (DA) neurons and the restoration of mitochondria [38].

Intriguingly, it was recently suggested that exosomes can improve various markers of PD pathophysiology and secrete various biomolecules, including microRNAs, which may serve as potential markers and modulators of various pathways underlying numerous disorders, including PD [2].

## Conclusion

Thus, based on the data mentioned, we can conclude that the treatment of rot-induced Parkinson’s disease (PD) model with exosomes is responsible for reprogramming and regenerating damaged neuronal cells, leading to repression of circRNA 2837. This frees the expression of miRNA-34b from its sponge-like effect. The expression of miRNA-34b reduces α-syn, clears Lewy bodies’ aggregates, and elevates TH activity. The freeing of miRNA-34b also results in the acceleration of parkin and DJ-1 production, which relieve the damaging effect on mitochondria and scavenge oxidative stress markers, contributing strongly to the symptoms and signs of PD. As a result, there is marked improvement in motor and behavioral manifestations of PD.

### Supplementary Information

Below is the link to the electronic supplementary material.

Supplementary file1 (MP4 8596 KB)

Supplementary file2 (MP4 48899 KB)

## Data Availability

The datasets generated during and analyzed during the current study are available from the corresponding author on reasonable request.

## Abbreviations

circRNAs: Circular RNAs
DA: Dopamin
DAn: Dopaminergic neurons
DJ-1: Protein deglycase
FACS: Fluorescence activated cell sorting
HSP70: Heat shock protein 70
miRNAs: microRNAs
mRNA: Messenger RNA
MSCs: Mesenchymal stem cells
ncRNAs: Non-coding RNAs
ND: Neurodegenerative
PD: Parkinson’s disease
ROS: Reactive oxygen species
rRNAs: Ribosomal RNAs
SNc: Substantia nigra cells
TEM: Transmission electron microscopy
TH: Tyrosine hydroxylase
α-SYN: α-Synuclein
